# Polymeric Gluten Proteins as Climate-Resilient Markers of Quality: Can LC-MS/MS Provide Valuable Information about Spring Wheat Grown in Diverse Climates?

**DOI:** 10.1021/acs.jafc.4c10789

**Published:** 2025-01-09

**Authors:** Sbatie Lama, Faraz Muneer, Antoine H.P. America, Ramune Kuktaite

**Affiliations:** a Department of Plant Breeding, Swedish University of Agricultural Sciences, Box 190, Lomma SE-23422, Sweden; b Wageningen Plant Research, Wageningen University and Research, Wageningen 6708 PB, The Netherlands; c Agricultural University of Iceland, H66H+9R9, Hvanneyrabraut, Hvanneyri 311, Iceland

**Keywords:** gluten polymer, spring wheat, glutenins, gliadins, severe climate impact

## Abstract

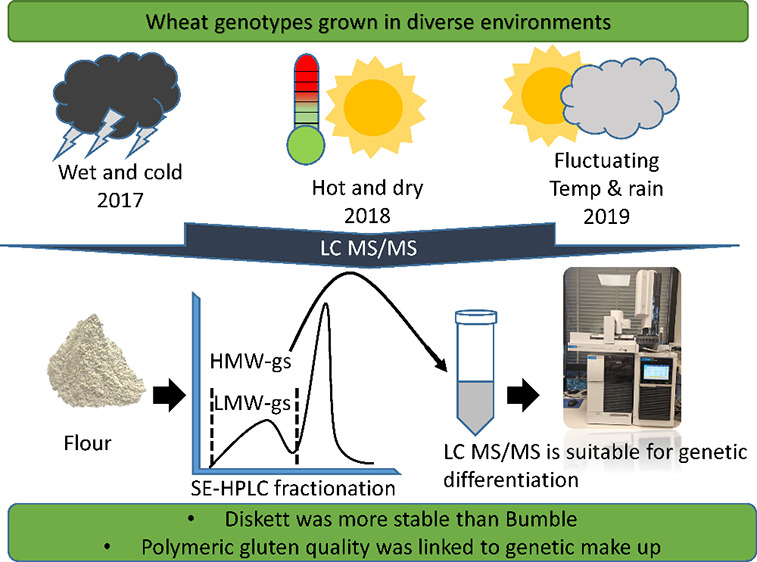

In this study, the impact of the varying environments, wet–cool (2017), dry–hot (2018), and fluctuating (2019), on two spring wheat genotypes, Diskett and Bumble, grown in field conditions in southern Sweden was studied. From harvested grains, polymeric gluten proteins were fractionated and collected using SE-HPLC and then analyzed with LC-MS/MS. Proteins and peptides identified through searches against the protein sequences of*Triticum aestivum* (taxon 4565) from the UniProtKB database showed 7 high molecular weight glutenin subunits (HMW-GS) and 24 low molecular weight glutenin subunits (LMW-GS) with different enrichment levels for both genotypes. Glu-B1 for HMW-GS and Glu D3 and m- and s-types for LMW-GS were dominated in both genotypes, and a small proportion of α-, γ-, and ω-gliadins were also present. A minor variation in HMW-GS and LMW-GS compositions was observed between the years, while small amounts of heat shock proteins were identified under the “dry–hot” period for Diskett. In conclusion, Diskett showed more stable and climate-resistant protein patterns in the studied varying climate as compared to Bumble. The study highlights the use of proteomics and LC-MS/MS for differentiation of wheat genotypes, although it shows low sensitivity in measuring the diverse environment impact on the polymeric proteins.

## 1 Introduction

Wheat is one of the most significant cereal crops across the globe and is used in a wide range of food products, where its protein components and their chemistry play a significant role in determining the end-product quality. The gluten protein quantitative and qualitative properties are regulated in a genetically strict manner and are sensitive to changes in the growing environment such as fluctuating temperatures and drought.^1,2^ Generally, wheat gluten proteins' molecular interplay is around 50% determined by genetics and 50% by growing environment.^3^ Therefore, an ongoing climate change is posing a major threat to the stability of gluten protein quality and wheat production worldwide, particularly during drought events and in the presence of unpredictable precipitation patterns, high temperatures, and increasing CO_2_ concentrations in the atmosphere.^4,5^

Wheat gluten proteins mainly consist of two types: gliadins and glutenins.^6,7^ Gliadins are monomeric proteins, cross-linked by the intramolecular disulfide bonds among the peptides, contributing mostly to viscosity and extensibility in processed food products.^9^ Glutenins are polymeric proteins that are further subdivided into two types depending on their molecular weight, i.e., high molecular weight glutenin subunits (HMW-GS) and low molecular weight glutenin subunits (LWM-GS).^8^ Glutenins contain both intra- and intermolecular disulfide bonding between peptides and proteins, contribute primarily to dough elasticity, and impart valuable viscoelastic properties to wheat dough and end-use quality.^10,11^ It is well established that baking quality is influenced by genotype, environment, and their interaction, primarily due to changes in the gluten polymer structure.^3^ A balance between both monomeric and polymeric gluten protein fractions is key for the specific functional properties of wheat bread-making quality,^12^ and this balance can be affected by the abiotic stresses during the crop production.^13,14^ Different gel-based and high-performance liquid chromatography (HPLC) methods have been widely used to identify the gluten protein composition of wheat grown in different environments,^1,11^ including those in, e.g., drought and heat.^15−17^ The use of the proteomics approach with the help of liquid chromatography (LC) coupled with mass spectrometry (MS)^18^ so far has been explored in few studies aiming to quantify the glutenin subunits (e.g., HMW-GS and LMW-GS).^19^ This highlighted different wheat proteins in seed and bread products.^20^ Previous studies have identified several peptides from entire gluten proteins using common enzymatic digestion methods,^21^ while other studies focused on gluten-enriched fractions.^18,22^ However, due to the complex gluten polymer chemistry, LC-MS/MS analysis of gluten proteins has been less explored especially regarding the role of HMW glutenins in determining the functionality.^23^

Plant proteome responds to drought stress factors, where different proteins play an important role in directly controlling the stress tolerance response and adaptation.^24,25^ Drought stress induces a number of morphological, physiological, and biochemical changes in all plant organs, which disrupts the relationship of sink and source plant organs.^25^ Despite a number of studies and crop models being adopted to understand plant–environment interaction in wheat, its impact on gluten protein quality, stability, and qualitative proteome under various abiotic stress conditions^5,26^ still remains a challenge. Modern high-throughput proteomics tools have highlighted the proteome responses of gluten proteins to various biotic and abiotic stresses^25,27^ and identified a magnitude of polypeptides and proteins that are synthesized in response to drought and heat using LC-MS/MS.^14,24,25,28,29^ However, a clear impact of the abiotic stresses on the polymeric gluten proteins is not fully understood, in particular how the fluctuating and excessive climates impact the polymeric gluten proteome. Therefore, more efforts are needed on the better exploration of LC-MS/MS use in mapping the gluten protein quality traits and their stability in diverse growing environments, including excessive drought and heat.

In this study, we investigated the gluten polymeric protein fraction from two spring wheat genotypes grown in field under three years (2017–2019), which varied highly in the environmental pattern, e.g., wet–cold (2017), dry–hot (2018), and fluctuating pattern (2019). The objective of this study was to investigate how the growing environment influences the composition of the wheat gluten polymer, and the focus was on the key proteins and associated peptides that are least impacted by the growing environment. We applied the integrated analytical approaches of SE-HPLC and LC-MS/MS to assess expected variations.

## 2 Materials and Methods

### 2.1 Growing Environments of Wheat Material

Two spring wheat genotypes, Diskett (referred to as G1) and Bumble (referred to as G2), were grown consecutively under 3 years, 2017 (wet–cool climate referred to as Y1), 2018 (dry–hot climate referred to as Y2), and 2019 (fluctuating climate referred to as Y3) by Lantmännen AB in Svalöv, Sweden (55°55′N and 13°07′E). Relevant growing environment information on precipitation (mm) and temperature during the wheat growing period under 2017, 2018, and 2019 (in comparison to the average precipitation and temperatures from the 2008 to 2022 year period) is shown in Figure S1 (Supporting Information).

### 2.2 Flour Milling

Mature dry wheat grains (2 g) of Diskett and Bumble were ground into flour using a grinder, Mixer Mill MM 400 (Retsch, Haan, Germany) at 20 Hz for 30 s. The milled wheat flour samples were freeze-dried for 24 h (CoolSafe Pro, Labogene, Lillero̷d, Denmark) prior to further analysis.

### 2.3 Protein Extraction for SE-HPLC Analysis

From each genotype, 50 mg (±0.1) of flour was dissolved in 1.4 mL of extraction buffer (0.05 M NaH_2_PO_4_, 0.5% SDS, and 1 mM *N*-ethylmaleimide (NEM), pH 6.9; NEM reactivity toward thiols was used to modify cysteine residues in protein) in a 1.5 mL Eppendorf tube. The samples were vortexed for 10 s in Whirli VIB 2 (Labassco, Sweden) and shaken for 10 min at 2000 rpm (IKA, Vibrax VXR, Germany). Thereafter, samples were sonicated for 45 s at an amplitude of 5 μ using an ultrasonic disintegrator (Soniprep 150, Sanyo, Japan) and immediately cooled in ice for 1 min followed by a second interval of sonication for 45 s in order to extract the so-called “unextractable” large polymeric gluten fraction. The extraction step included both the extraction buffer and the sonication in order to extract a large polymeric gluten fraction containing both “extractable” and “unextractable” protein fractions (retention time interval 8–13.5 min).^16^ After sonication, the samples were centrifuged for 30 min at 9600*g*. The supernatant was collected in glass vials and heated at 80 °C for 2 min (to inactivate proteases) in a water bath as described by Islas-Rubio et al.^30^ Immediately after heating, the vials were cooled in an ice bath for 1 min and proceeded for sodium dodecyl sulfate-polyacrylamide gel electrophoresis (SDS-PAGE) and SE-HPLC analysis.

### 2.4 SDS-PAGE Analysis

SDS-PAGE analysis was performed to determine the protein composition of the wheat flour according to Nynäs et al.^31^ with some modifications. From the extracted gluten proteins from the HPLC step extraction, 5 μL was mixed with 7.5 μL of a sample buffer (Novex Bolt LDS sample buffer 4%, Invitrogen, Thermo Fisher Scientific, Massachusetts, USA), 3 μL of sample reducing agent (Novex Bolt 10x sample reducing agent) and 14.5 μL of Millipore (MQ) water in an Eppendorf tube. The samples were heated in a water bath for 4 min at 92 °C and cooled down in an ice bath, and 15 μL of sample was loaded on a precast gradient mini-gel (Novex Bolt 4–12% Bis–Tris Plus gel) using MES SDS running buffer (Bolt 20x MES). A protein ladder of 5 μL (SeeBlue Plus2 Pre-stained, Standard, Invitrogen, Thermo Fisher Scientific, Massachusetts, USA) was used. The gel was run for 30 min at 130 V and afterward washed three times with MQ water. The gel was stained with approximately 20 mL of stain (GelCode Blue Safe Protein Stain, Thermo Fisher Scientific, Massachusetts, USA) for 15 min while shaking gently and destained with MQ water overnight (with the change of water a few times).

### 2.5 Polymeric Protein Fractionation by SE-HPLC

**Figure 1:**
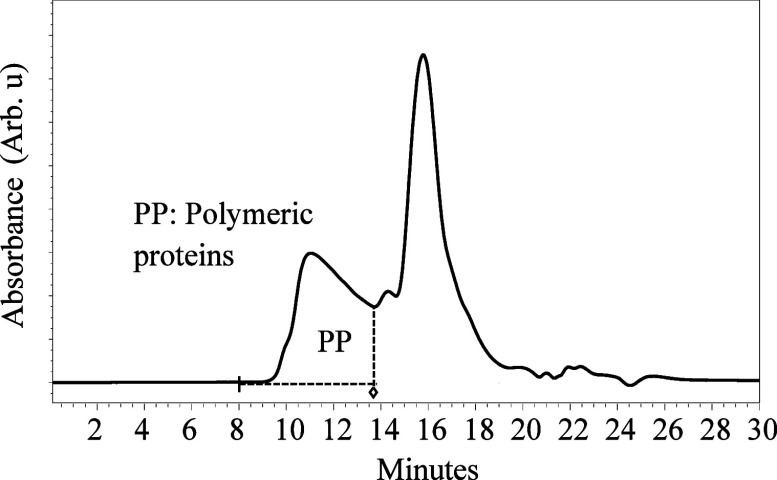
SE-HPLC chromatogram of the polymeric protein fraction extracted with SDS-NaH_2_PO_4_ and NEM from wheat flour of Diskett from 2017, which was used in the LC-MS/MS study. Retention times to collect the polymeric proteins were 8–13.5 min.

For fractionation of gluten protein with SE-HPLC, 20 μL was injected into the Waters Alliance 2695 Separations Module HPLC system (Waters, Massachusetts, USA) and separated for 30 min using a BioSep SEC-4000 Phenomenex SE-HPLC column at an isocratic flow of 0.2 mL/min (50% acetonitrile, 0.1% TFA; 50% H_2_O, 0.1% TFA) at 23 °C with the aim to collect the polymeric gluten proteins at the retention time interval 8–13.5 min (Figure 1). The polymeric proteins from each injection were collected by a fraction collector (Waters Fraction Collector III) connected to the SE-HPLC system. From each replicate of extracted protein, polymeric protein fractions were collected from 20 injections in 15 mL centrifuge tubes. Protein extractions for each genotype grown in each year were conducted in triplicate (R1, R2, and R3).

### 2.6 LC-MS/MS Analysis

#### 2.6.1 Sample Preparation

For filter-aided sample preparation (FASP), a Microcon-10 kDa centrifugal filter unit with Ultracel-10 membranes (ultrafilter) were used, and for solid phase extraction (SPE), Pierce C18 Spin Tips and 100 μL columns were used according to Erde et al.^32^ and Ostasiewicz et al.^33^ The FASP ultrafilter, formic acid (FA, purity 98–100%), and iodoacetamide (product number RPN6302) were obtained from Merck Millipore (Massachusetts, USA). The C18 Spin Tips, trifluoroacetic acid (TFA, purity ≥99), dithiothreitol (DTT, purity 99%), and chymotrypsin endoproteinase (product number 90056) were obtained from Thermo Fisher Scientific (Massachusetts, USA). Urea (purity ≥99%) was obtained from Duchefa Biochemie (Haarlem, The Netherlands), and acetonitrile (ACN, purity 100%) was obtained from VWR (Pennsylvania, USA).

For FASP, the polymeric gluten protein fraction collected via SE-HPLC was concentrated in 2 mL Eppendorf tubes using a SpeedVac (Savant SVC100, Thermo Fisher Scientific, Massachusetts, USA). Polymeric gluten protein fractions from 20 injections, representing one replicate, were concentrated to a final volume of 0.5 mL. To prevent protein precipitation, 100 μL of 8 M urea was added to the concentrated protein solution. The concentration of the proteins was measured using a NanoDrop (DeNovix DS-11 FX, Labgene Scientific, Switzerland) at protein absorbance wavelength A280 (with a protein concentration of 0.1 mg/mL). The concentrated protein fractions were kept in an ultrafilter unit and stored overnight at 4 °C. The following day, the samples were centrifuged at 9600*g* for 15 min. To remove the SDS, 15 μL of 2 M urea and 43.75 mM DTT were added to the membrane and centrifuged at 9600*g* for 15 min. This step was repeated twice.^34^ Afterward, 465 μL of 20 mM iodoacetamide was added in the ultrafilter unit with proteins to alkylate free SH groups in the proteins, followed by centrifuging at 9600*g* for 20 min.^33^ The protein concentrate on the membrane was complemented with 50 μL of 8 M urea, 5 mM DTT, 250 μL of ammonium bicarbonate (100 mM), and 150 μL of MQ water to achieve the final concentration of urea of 1 M. The membrane was centrifuged at 9600*g* for 15 min, and all the liquid that passed through the filter after centrifugation was discarded.

The ultrafilter unit containing the protein concentrate was transferred to a new fresh Eppendorf tube, and 100 μL of 100 mM ammonium bicarbonate containing 1 μg of chymotrypsin was added to the membrane. The membrane was further incubated (Thermomixer Comfort, VWR, Pennsylvania, USA) at 30 °C with continuous shaking at 300–600 rpm for 4 h followed by addition of 50 μL of ammonium bicarbonate (100 mM) followed by incubation for 5 min at 300–600 rpm. The membrane was centrifuged at 11,000 rpm for 20 min, and eluted peptides (∼150 μL) were collected in an Eppendorf tube. For the second elution, 50 μL of ammonium bicarbonate was added to the membrane and stored overnight at 4 °C. The next day, the membrane was incubated at 30 °C at 500 rpm for 30 min followed by 20 min centrifugation at 11,600*g* to collect eluting peptides from the polymeric gluten proteins. To the extracted peptides, 1.5 μL of 10% TFA was added to acidify and stop the reaction of the chymotrypsin enzyme.

#### 2.6.2 SPE

The SPE column (C18 Spin Tips and columns) was washed with 1 mL of 95% ACN and equilibrated with 1 mL of 2% ACN and 0.1% TFA according to the method of Erde et al.^32^ Subsequently, the digested peptides were added to the columns, and after washing with 1 mL of 2% ACN and 0.1% FA, the peptides were eluted with 1 mL of 50% ACN and 0.1% FA. The eluted peptide fraction was dried in a SpeedVac and dissolved in 100 μL of 2% ACN and 0.1% FA.

#### 2.6.3 Untargeted LC-MS/MS-Orbitrap Analysis

The extracted digested peptides from the polymeric gluten proteins were injected and separated with a Waters M-Class UPLC system (Waters, Massachusetts, USA) online connected to a Q Exactive Plus mass spectrometer (Thermo Fisher Scientific, Massachusetts, USA). The peptides were first collected on a trap column (20 cm × 0.15 mm PepSep C18 Trap, PepSep, Marslev, Denmark) and subsequently separated on a 10 cm × 75 μm analytical C18 column (PepSep, Marslev, Denmark) with a 45 min gradient of 6 to 30% acetonitrile in 0.1% FA followed by a column clean (80% acetonitrile 0.1% FA) and restore phase (2% acetonitrile and 0.1% FA all at a flow rate of 200 μL/min) according to the method described by Geisslitz et al.^35^ The eluted peptides were electrosprayed into the Q Exactive mass spectrometer using a Flex Ion nanoESI source at +2.4 kV. Mass spectrometry (MS) and MS/MS spectra were collected in top 10 DDA mode selecting charge 2, 3, or 4 ions within *m*/*z* range of 400–1500 for MS spectra and autorange for MS/MS spectra.^35^

#### 2.6.4 Data Evaluation for LC-MS/MS

The peptides and proteins of the gluten polymeric fraction digests analyzed by LC-MS/MS-Orbitrap were identified using MaxQuant (version 1.6.17)^36,37^ by searching the MS data against the collection of protein sequences of *Triticum aestivum* (taxon 4565) from the UniProtKB database downloaded on 19th May 2021 containing 143,500 entries using the search engine Andromeda with included contaminant protein sequences and the reverted decoy database. Carbamidomethylation or NEM modification of cysteine, oxidation of methionine, and acetylation of the proteins N-terminus were selected as potential variable modification. Bovine pancreas chymotrypsin was used as the proteolytic enzyme that specifically cleaves at the carboxyl side of tyrosine, phenylalanine, tryptophan, and leucine amino acids.

Match between runs was enabled. Protein quantification was done on LFQ values based on the razor peptides, with modified peptides included. The results were filtered for minimal and maximum peptide lengths of 7 and 40, respectively, with 1% peptide and protein false discovery rate.

### 2.7 Statistical Analysis

The statistical analyses were performed using the software R (https://www.r-project.org/). The statistical evaluation was performed on the proteins where three replicates from LC-MS/MS runs of the gluten proteins were used for principal component analysis (PCA) to evaluate variation in the protein intensity across different genotypes (G) and years (Y). For PCA, Log2-transformed LFQ intensity values of all the “Leading razor protein” were used (File S1, sheet: *peptides*, column: *Leading razor protein*); all undefined values were replaced with the minimal value 15 (due to Log2 of 0 is undefined). Hierarchical clustering of the protein intensities (Log2-transformed LFQ intensity values) of the polymeric gluten proteins, HMW-GS, and LMW-GS was performed using a heat map package in R. To investigate peptide-level variations in HMW-GS and LMW-GS (and gliadins) between the years (Y) for each genotype (G), we conducted Student’s *t* test (*p* < 0.05) for the LFQ intensity values of the peptides; LFQ intensity values of 0 were replaced with the lowest LFQ intensity within each replicate (e.g., for genotype G1 in year Y1, replicate R1, all 0s were replaced with the lowest LFQ intensity for G1Y1R1, which is 39158). Fold changes were calculated as the Log2 ratio of the average LFQ intensity of three replicates for one genotype-year to the corresponding average of another year (e.g., Log2[Avg(Y1)/Avg(Y2)] for G1). Adjusted *p*-values were calculated using the Bonferroni correction method.

## 3 Results and Discussion

### 3.1 Characterization of Wheat Material by SDS-PAGE Gel Electrophoresis

The SDS-PAGE electrophoresis of total gluten protein extracted from Diskett and Bumble flours has indicated the HMW-GS composition, where both have an allelic pair of Dx5+Dy10 subunits, combined with Ax2* in Diskett and Ax1 in Bumble, respectively (allele designations according to Payne and Lawrence 1983)^38^ (Supporting Information, Figure S2). Some differences in protein bands of the gliadin/LMW subunit in Bumble grown in 2018 and 2019 compared to 2017 and Diskett were observed (Supporting Information, Figure S2). The subunits were most likely related to the C-type LMW-GS (30–40 kDa in size) as a similar trend was found in other studies due to drought stress.^39−41^ Since the HMW-GS combination of Dx5+Dy10 is primarily associated with high gluten strength and superior baking performance,^42^ the wheat material in this study was expected to deliver a similar outcome from the genetic background. In addition, the HMW-GS subunits Ax2* and Ax1 on Glu A1 are also known being positively related with the strong bread-making properties, e.g., bread volume,^43^ sedimentation volume, and dough mixing time, indicating the material's strong bread-making properties.^44,45^ Indeed, the HMW-GS subunit Dx5+Dy10 combination is highly preferred (containing an extra cysteine compared to the HMW-GS subunit 2) from the breeding and bread baking industry perspectives in order to deliver desired polymerization and baking performance^28^ for the Swedish wheat product market.

### 3.2 Polymeric Protein Sample Preparation and Analysis by LC-MS/MS-Orbitrap

#### 3.2.1 Polymeric Protein Preparation Prior to LC-MS/MS Analysis

The polymeric gluten protein fraction, the main determinant of gluten strength, was extracted using a common SDS-phosphate buffer with addition of *N*-ethylmaleimide (NEM) and sonication, and this fraction was further separated using SE-HPLC (at retention time interval 8–13.5 min, Figure 1) (*N*-ethylmaleimide was used for differentiation of unbound/free cysteine residues from disulfide bridges in the gluten polymer). The polymeric gluten protein fraction after separation by SE-HPLC was chemically reduced and alkylated to primarily unfold and break the disulfide cross-links between the gluten protein polypeptide chains, and this fraction was further subjected to chymotrypsin digestion where the proteins were further converted into soluble peptides, which were later analyzed by LC-MS/MS. In this study, the major focus was on the main constituents of the gluten polymer, HMW-GS and LMW-GS, as well as gliadins (as potentially trapped protein in the large polymer network).

#### 3.2.2 HMW-GS and LMW-GS Composition Characterization by LC-MS/MS

The digests from the polymeric gluten protein fractions of Diskett and Bumble were subjected to LC-MS/MS analysis, and the data obtained were processed using MaxQuant software. The proteins (and peptides) were identified by searches against the collection of protein sequences of *T. aestivum* (taxon 4565) from the UniProtKB database, which included more than 134,500 entries (as of May 2021). In total, 465 protein groups were identified based on 1144 peptide identifications (decoys and contaminants removed) (Supporting Information, Data File S1). From the table of identified proteins, HMW-GS and LMW-GS were filtered, and the LFQ intensity (on log2 scale) is presented as a heat map in Figure 4. The proteins with 0 LFQ intensity were excluded from the analysis.

### 3.3 Relationship of Protein Expression between Genotypes and Production Years

**Figure 2:**
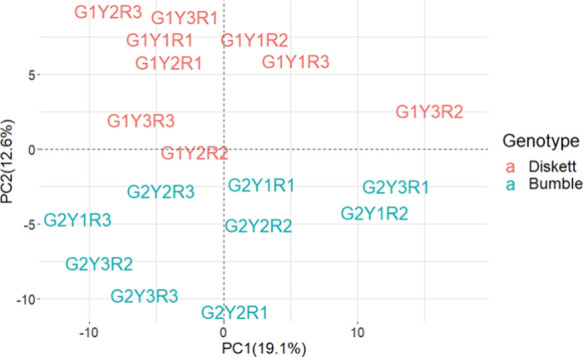
Principal component analysis (PCA) plot of variation in the collective protein expression in the isolated polymeric gluten fraction (Log2 value of LFQ intensity) of two genotypes, Diskett (G1) and Bumble (G2), studied for 3 years (2017—Y1, 2018—Y2, and 2019—Y3). The replicates are designated as R1, R2, and R3.

PCA was performed on the Log2 value of LFQ intensities of the proteins to investigate the relationship of the total proteins' enrichment levels between genotypes and in respective production years (Figure 2). The PCA results showed that PC1 and PC2 explained 19.1 and 12.6% variability, respectively (Figure 2). Total proteins expressed showed a clear distribution between genotypes, where Diskett and Bumble were clustered along each side of the plot (Figure 2), whereas the distribution of Diskett across different production years showed a closer association within replicates from harvest years 2017 and 2018, and a larger variation was observed among samples harvested in 2019 (Figure 2). In Bumble, for the sample distribution for 2018, a year with strong heat and drought, the impact during plant growth was relatively different from those for 2017 and 2019 (Figure 2). In this study, a large variation between the replicates for Bumble might partially be explained by the greater genotype sensitivity to the seasonal variation, although the polymeric protein fraction isolation and preparation for LC-MS/MS analysis should be in more detail evaluated and monitored. Important to mention is that, in our previous studies, the genotype grown in severe heat and drought conditions required an inactivation of proteases to avoid degradation of polymeric protein fraction during the protein extraction.^16,17^

The PCA clearly suggested that the two genotypes differ in the protein expression level (Figure 2); however, the production year had a significant impact on the variation of total protein composition of the polymeric gluten fraction, especially among Bumble samples. This variation can be partially explained by diverse growing seasons with great variation in average temperature and precipitation patterns (Supporting Information, Figure S1). Specifically, the year 2017 was relatively cooler with higher precipitation levels; 2018 was dry–hot, whereas 2019 was with high fluctuating temperatures during June–August (Supporting Information, Figure S1). In this study, Diskett showed a relatively more stable response to changing growth environments in 2017 (wet and cold) and 2018 (dry and hot), as shown by a close association of the samples compared to Bumble.

### 3.4 Composition of Polymeric Protein Fraction

**Figure 3:**
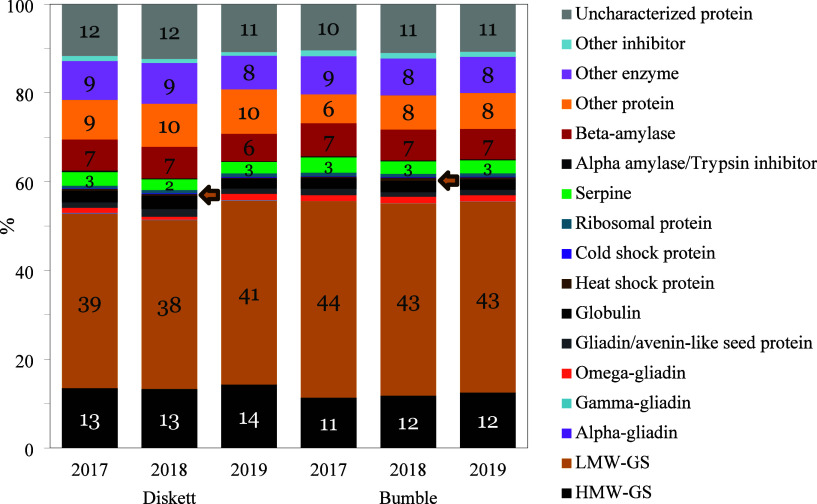
Composition of expression of different proteins found in the enriched polymeric protein fraction of the two genotypes (Diskett G1 and Bumble G2) in 3 years (2017, 2018, and 2019).

The polymeric protein fractions of both genotypes contained multiple types of proteins (besides HMW-GS and LMW-GS), including gliadins, serpins, and multiple types of enzymes (Table 1). Summarized and normalized LFQ intensities per protein class presented relative to the total LFQ value are shown in Figure 3. We observed rather similar amounts of HMW-GS between the genotypes ranging in intensity from 11 to 14%, although somewhat greater amounts (1–2%) were noted for Diskett. In terms of LMW-GS, slightly higher amounts were observed for Bumble compared to Diskett (similar to that indicated in SDS-PAGE gel, Supporting Information, Figure S2). In both HMW-GS and LMW-GS patterns, a minor variation in Diskett between the years was observed (Figure 3). The polymeric fraction of wheat gluten proteins consists of mostly HMW-GS and LMW-GS subunits, and the gliadins are found in the monomeric gluten fractions.^11^ From a large proportion of other proteins, it is important to mention that higher enrichment levels of heat shock protein HSP90 (A0A3B6MTT2) were observed for Diskett in hot–dry 2018 (Supporting Information, Data File S2, comparison between G1G2Y2). In comparison, during 2019, Bumble showed higher enrichment levels of ST1 domain proteins (A0A3B6QFL1) compared to Diskett, which are co-chaperon of HSP90 and HSP70 proteins known to play a role to fine-tune the heat stress response in plants (Supporting Information, Data File S2, comparison between G1G2Y3).^46^

**Table 1:** Protein Classification and Peptide (LFQ Intensity × 10^6^) Significantly Differential Abundances in Diskett (G1) and Bumble (G2) from Different Growing Years^a^

protein class	protein accession	MS/MS count	LFQ intensity (10^6^)		peptide sequence	score	*p* value	referenced found in other studies/gene clone
			**G1Y1**	**G1Y2**				
HMW-GS Dy10-M328sf	A0A2L1K3K4	4	37.84 (±15.46)	0.31 (±0.18)	PGQASPQQPGQGQQPGKW	347.99	0.01	Glu-B1
LMW-GS	K7WV92	76	0.30 (±0.27)	36.08 (±10.6)	EAIRAIIF	143	0.02	Glu 3
LMW-GS D1	Q00M56	51	54.95 (±10.24)	112.76 (±9.4)	VHPSILQQLNPCKVF	162.93	0.02	Lee et al., 2016, Zhao et al., 2006
D-type LMW-GS	B6ETR9	42	43.71 (±6.13)	72.87 (±12.8)	PLQPQQPFPLRPQQPF	223.78	0.04	
S-type LMW-GS L4-36	Q6J161	7	20.29 (±3.59)	0.31 (±0.18)	SQQELPILPQQPPF	172.1	0.02	
Alpha-gliadin PSQ2	A0A0K0VHI4	79	204.17 (±8.94)	240.35 (±11.4)	IPPYCSTTIAPF	140.98	0.02	
Alpha/beta-gliadin	A0A0K2QJD8	39	89.65 (±11.32)	139.87 (±15.9)	LPQLPYPRPQPF	230.09	0.02	
Alpha/beta-gliadin	A0A0E3Z506	12	16.556 (±3.79)	0.31 (±0.18)	PQPQLPYPQPQPF	145.72	0.02	
Gamma gliadin	D0ES79	4	0.30 (±0.27)	290.04 (±310.6)	LQQQMNPCENY	50.108	0.02	
Z-like serpin/chymotrypsin inhibitor	Q9S8N3	193	963.52 (±172.84)	346.56 (±14.9)	QARPPSVMDFIADHPFLF	339.87	0.01	
Serpin-Z2A	A0A3B6KQL2	55	151.44 (±14.65)	101.98 (±7.7)	LLDGSSIQTPF	123.16	0.01	GluD3--3
Serpin-Z1C;Serpin-N3.7	Q9ST58	50	357.24 (±45.51)	236.50 (±37.5)	VLADASSAGGPHVAF	179.36	0.04	Vensel et al., 2014
Serpin-Z2A	A0A3B6KQL2	30	559.98 (±58.19)	347.11 (±47.7)	VLADASSVGGPRVAF	231.07	0.02	
SERPIN domain-containing protein	A0A3B6KQL2	24	451.92 (±35.95)	284.61 (±27.7)	ILLPEARSGIW	128.36	0.01	Vensel et al., 2014 (Glu-B1)
			G1Y1	G1Y3				
HMW-GS	A0A0X9BSF8	43	427.72 (±48.24)	786.33 (±106.4)	LSVTSPQQVSY	182.4	0.007	
HMW-GS	A0A0X9BSF8	9	57.50 (±6.50)	109.49 (±20.9)	LTSPQQSGQW	143.37	0.022	
D-type LMW-GS	B6ETR9	55	1719.1 (±342.3)	3986.67 (±997.6)	ARQLNPSNKELQSPQQSF	422.25	0.021	
Alpha-gliadin	K7XRA8	1	63.76 (±6.27)	112.18 (±6.2)	LQPQLPY	116.87	0.005	
Gamma-gliadin	B6UKN7	74	422.91 (±33.21)	604.66 (±33.6)	ASIVAGIGGQ	199.02	0.007	
Z-like serpin/chymotrypsin inhibitor	Q9S8N3	193	963.52 (±172.8)	351.92 (±48.9)	QARPPSVMDFIADHPFLF	339.87	0.003	
Serpin-Z1B	P93693	12	44.03 (±2.41)	32.19 (±3.6)	KGAWTDQFDSY	120.15	0.037	Vensel et al., 2014
			G1Y2	G1Y3				
X-type HMW-GS DX5	Q0Q5D2	65	2166.0 (±310.8)	3299.17 (±449.9)	YPGQASPQRPGQGQQPGQGQQGY	460.38	0.040	Vensel et al., 2014
HMW-GS	A0A0X9BSF8	43	449.62 (±25.70)	786.33 (±106.4)	LSVTSPQQVSY	182.4	0.014	
**Glu-B1-1b HMW-GS**	**Q42451**	**42**	**477.97 (210.44)**	**35.18 (13.48)**	**QQPGQGQQPGQGQQSGQGQQGQ QPGQGQRPGQGQQGY**	**324.96**	0.0282	
HMW-GS 1By9; By8	Q03871	2	0.31 (±0.18)	5.67 (±2.4)	YPGETTPLQQLQQVIF	84.198	0.029	
D-type LMW-GS	B6ETR9	21	0.31 (±0.18)	181.78 (±216.5)	PQQPHQPQQPYPQQQPY	313.04	0.009	
Omega-gliadin	A0A060N0S6	23	123.56 (±41.02)	362.09 (±154.9)	ARELNPSNKELQSPQQSF	244	0.045	
			G2Y1	G2Y2				
Glu-B1-1b HMW-GS X-type Bx7	Q42451	9	4.81 (±2.64)	0.43 (±0.1)	LQPGQGQQGY	163.72	0.016	
LMW-GS A3-2	D3UAL5	12	202.40 (±77.04)	0.43 (±0.1)	SQQQQPPFSQQQQPPF	138.42	0.0000831	Wang et al., 2010, Lee et al., 2016
Alpha/beta-gliadin A-II	A0A0E3Z6M2	257	321.94 (±13.60)	253.00 (±10.1)	IPPYCTIAPF	170.17	0.004	
Alpha-gliadin	A0A1W6C2K0	41	67.88 (±6.68)	45.86 (±6.5)	IPPYCTIAPVGF	153.73	0.033	
Gamma-gliadin-B1	P21292	14	0.69 (±0.11)	64.06 (±11.3)	ANIDAGIGGQ	218.04	0.0000133	
Serpin-Z1C; Serpin-N3.7	Q9ST58	50	395.83 (13.03)	253.75 (±38.2)	VLADASSAGGPHVAF	179.36	0.048	Vensel et al., 2014
Serpin-Z2A	A0A3B6KQL2	30	647.07 (±60.29)	317.14 (±52.6)	VLADASSVGGPRVAF	231.07	0.014	
Serpin-Z2A	A0A3B6KQL2	27	243.67 (±30.06)	166.43 (±21.5)	LIREDTSGVVLF	120.76	0.042	Vensel et al., 2014
Serpin-Z2A	A0A3B6KQL2	24	593.88 (±89.30)	258.34 (±56.7)	ILLPEARSGIW	128.36	0.014	
			G2Y1	G2Y3				
HMW-GS	A0A0X9BSF8	14	47.53 (±16.14)	0.48 (±0.3)	YPTSPQQSGQGQQPGQW	219.92	0.0055	Vensel et al., 2014
HMW-GS	A0A0X9BSF8	8	61.33 (±16.44)	0.48 (±0.3)	YPTSQQQPGQGPQPGQW	130.36	0.0077	Vensel et al., 2014
HMW-GS	A0A0X9BSF8	4	66.714 (±32.48)	0.48 (±0.3)	YPTSPQQSGQGQQLGQGQQGY	296.1	0.0029	Vensel et al., 2014
S-type LMW-GS L4-36, LMW-D8	Q6J161	4	72.53 (±10.95)	124.12 (±7.9)	GVGTQVGAY	161.9	0.0215	Vensel et al., 2014
Alpha-gliadin	A0A0E3Z576	32	54.50 (±2.04)	0.87 (±0.8)	IPPYCTIAQVGIF	154.12	0.0286	
Alpha/beta-gliadin	A0A0E3Z506	249	2109.3 (±435.5)	1143.81 (±235.9)	IPPYCTIAPVGIF	185.55	0.0497	
Alpha/beta-gliadin	A0A1W6C2K0	41	67.88 (±6.68)	51.71 (±4.4)	IPPYCTIAPVGF	153.73	0.0472	
Gamma-gliadin	B6UKN1	74	774.12 (±53.64)	1183.53 (±63.1)	VRPDCSTINAPF	126.07	0.0032	
Gamma gliadin-D1	B6UKP0	43	528.59 (±60.88)	733.61 (±73.6)	ASIVVGIGGQ	175.44	0.0382	
Gamma-gliadin	P21292	14	0.69 (±0.11)	72.21 (±30.1)	ANIDAGIGGQ	218.04	0.0035	
Serpin-Z1B	P93693	21	1734.53 (±39.9)	1182.67 (±120.2)	QTKAAEVTTQVNSW	282.61	0.029	
			G2Y2	G2Y3				
HMW-GS Dy10-m328SF	A0A2L1K3K4	12	41.47 (±11.09)	0.48 (±0.3)	YPGVTSPRQGSYY	133.76	0.0098	Vensel et al., 2014
HMW-GS	A0A0X9BSF8	4	32.412 (±3.80)	0.48 (±0.3)	YPTSPQQSGQGQQGYDSPY	292.57	0.015	Vensel et al., 2014
Alpha-gliadin	A0A0E3Z576	32	15.98 (±6.80)	0.87 (±0.87)	IPPYCTIAQVGIF	154.12	0.039	
Gamma-gliadin	B6UKN1	74	741.74 (±34.02)	1183.53 (±63.1)	VRPDCSTINAPF	126.07	0.0007	
Gamma-gliadin	B6UKM7	13	150.01 (±9.55)	185.16 (±5.8)	CSTIRAPF	137.18	0.026	
Serpin 2;Serpin 4	A0A3B6TLW2	2	0.43 (±0.13)	14.03 (±10.8)	VENVTTGLIREILPEGSIDY	81.448	0.020	

a Statistical significance was set at *p* < 0.05 using Student *t*-test; Y1, Y2, and Y3 represent 2017, 2018, and 2019, respectively; HMW-GS and LMW-GS are highlighted in bold font.

In this study, a small proportion of alpha-, gamma-, and omega-gliadins were also identified in the polymeric gluten fraction (Figure 3), which is in agreement with our earlier study showing omega-gliadins trapped in the gluten polymeric network.^11^ Furthermore, the alpha-, gamma-, and omega-gliadins, as well as serpins, globulins, amylase trypsin inhibitors, and β-amylases, have been previously reported in SDS-soluble and insoluble polymeric gluten proteins.^29,47^ The presence of small amounts of gliadins in the polymeric gluten can be explained by gliadins having an odd number of cysteines, which can bind to the polymeric network where they are either linked together or with glutenins and act as chain terminators.^8^ In a previous study, minor amounts of alpha-/beta-, gamma-, and omega-gliadins, together with serpins, triticins, and globulins, were found incorporated in a reduced HMW-GS fraction.^29^

Serpins naturally present in wheat flour can be extracted up to 40% with buffer or salt solutions, and the remaining 60% are bound and require DTT treatment to become soluble. This suggests that there are molecular interactions with individual serpins or between serpin and gluten proteins in the HMW protein fraction.^48^ Serpins are also reported to contain either one, two, or three cysteine residues, which suggests that serpins are either covalently bonded with glutenin polymer and they may also serve as chain terminators similar to gliadin.^29^

From our study, it should be noted that of all 146 identified peptides containing cysteine, the majority (103) were alkylated by iodoitamide, and fewer (54) were identified with NEM modification (not shown). The only identified peptides in the polymeric gluten fraction with an NEM-modified cysteine were (MET)RCIPGLERPW, with the N-terminus of P94021 and V9P6Q7, respectively. This N-terminally located cysteine is spatially separated from the other seven cysteines embedded in the repetitive domains of the LMW sequence. As such, the N-terminal cysteine appears to be less involved in S–S disulfide bridges of intra- or intermolecular protein interactions.

#### 3.4.1 HMW-GS and LMW-GS

**Figure 4:**
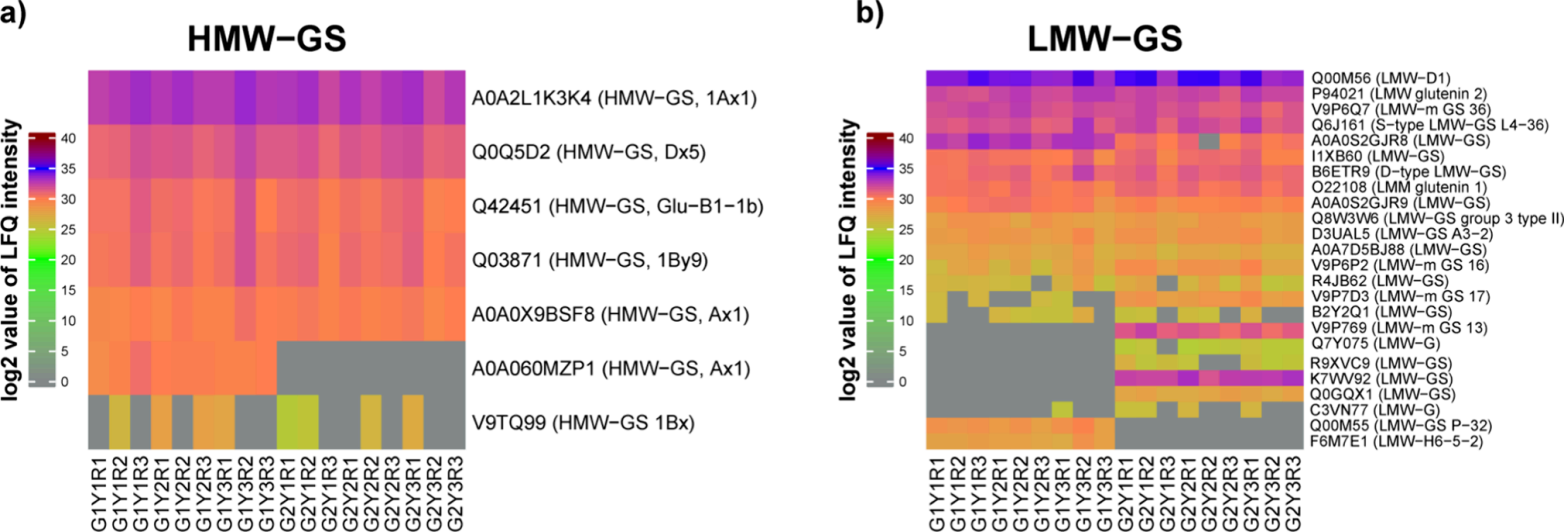
Heat map of the log2 value of LFQ intensity of filtered (a) high molecular weight glutenin subunits (HMW-GS) and (b) low molecular weight-glutenin subunits (LMW-GS) studied by LC-MS/MS of Diskett (G1) and Bumble (G2) grown under 3 years (2017 (Y1), 2018 (Y2), and 2019 (Y3). Three replicates (R) are designated as R1, R2, and R3. Triticum proteome derived from UniProtKB was used to identify the proteins.

From the analysis, 7 HMW-GS and 24 LMW-GS were found, although a large variation in their enrichment levels was observed for both Diskett (G1) and Bumble (G2) (4). In regard to the HMW-GS composition, a variation for Diskett was observed between the environments studied with relatively higher intensities for the HMW-GS such as Glu-B1, X-type, Glu-B1-1b, and Glu-1By9 during the fluctuating season (2019) compared to wet-cool (2017) and dry-hot (2018) seasons (Figure 4a). No clear impact of the varying climate for the HMW-GS composition was observed for Bumble (Figure 4a). For LMW-GS, the most dominating intensities of the top seven proteins were found for GluD3 and S-type proteins (Figure 4b). More proteins were found for Bumble than for Diskette (Figure 4b). To conclude, clear major differences in the protein composition were observed between the studied genotypes (Figure 4).

The peptide abundance sequences corresponding to different protein types including HMW-GS, LMW-GS, and gliadins (all of which are known as components of gluten polymer) were identified from the MS/MS spectra, as shown in Table 1. Among the seven HMW-GS, *x*-type (A0A2L1K3K4, Q0Q5D2, and V9TQ99) and *y*-type (Q03871) glutenins were identified (Figure 4a). In UniProtKB, Q0Q5D2 showed 100% similarity to P10388, which is a Dx5 protein. Although no match was found for Dy10, our SDS-PAGE result showed that Dx5+Dy10 were present in both genotypes (Supporting Information, Figure S2). In this study, Ax2* was found in Diskette and Ax1 for Bumble, as was also confirmed by SDS-PAGE (Supporting Information, Figure S2). In addition, the two HMW-GS proteins, *x*-type and *y*-type subunits, were identified as Q42451 (Bx) and Q03871 (By9) (Figure 4a). Among all the HMW-GS types of proteins, A0A060MZP1 (90% similar to Ax1) was found unique to Diskett, although the protein abundance (LFQ intensity) was much lower as compared to other identified proteins such as A0A2L1K3K4. Protein V9TQ99 was intermittently identified for both genotypes but at low intensities (Figure 4a). Given the variability observed in the replicates, we chose to present individual replicate data rather than means to better capture the stability and reproducibility of the method. The variability within replicates that we observed for V9TQ99 underscores this point.

In regard to LMW-GS, 24 predominantly present protein types including s-type, m-type, and proteins from all three A3, B3, and D3 loci regions were identified (Figure 4b). Among them, the highest amount of protein Q00M56 from Glu D1 was present in both genotypes in all seasons (Figure 4b). In addition, three proteins from P94021 (LMW-glutenin 2), V9P6Q7 (LMW-GS m 36), and Q6J161 (S-type LMW-GS L4-36) were also identified in the samples, although in lower intensities as compared to Glu D1 protein types (Figure 4b).

The effects of Glu-1 and Glu-3 glutenin loci highlighted in this study and their interaction are known to impact the dough rheological properties, such as resistance and extensibility, and play an important role in the baking quality of different wheat products.^49^ Also, the studied wheat genotypes include the allelic variants of Glu-B3 indicating strong rheological properties and bread-making quality.^50^

In addition, LMW-glutenin 2 is known to be associated with good pasta-making quality in durum wheat.^51^ In this study, the protein A0A0S2GJR8 belonging to the LMW-GS family showed notably higher intensity in Diskett as compared to Bumble (Figure 4b), suggesting a superior bread-making performance, while the other proteins, V9P769 (LMW-m GS 13), Q7Y075, R9XVC9, K7WV92, and Q0GQX1, as types of LMW-GS, were uniquely observed for Bumble (Figure 4b). To our understanding, the proteins that are present in the polymeric gluten fraction separated by SE-HPLC in this study include not only HMW-GS and LMW-GS protein groups largely associated with gluten strength but also gliadins corresponding to an extensible behavior.^50^ In our earlier experiments confirmed in dough,^11^ even smaller proteins such as albumins (or enzymes) were trapped in the gluten polymer, which can also be referred to the characterized species indicated in Figure 4 and Table 1.

### 3.5 Variation in Enriched Proteins in the Polymeric Gluten Fraction

**Figure 5:**
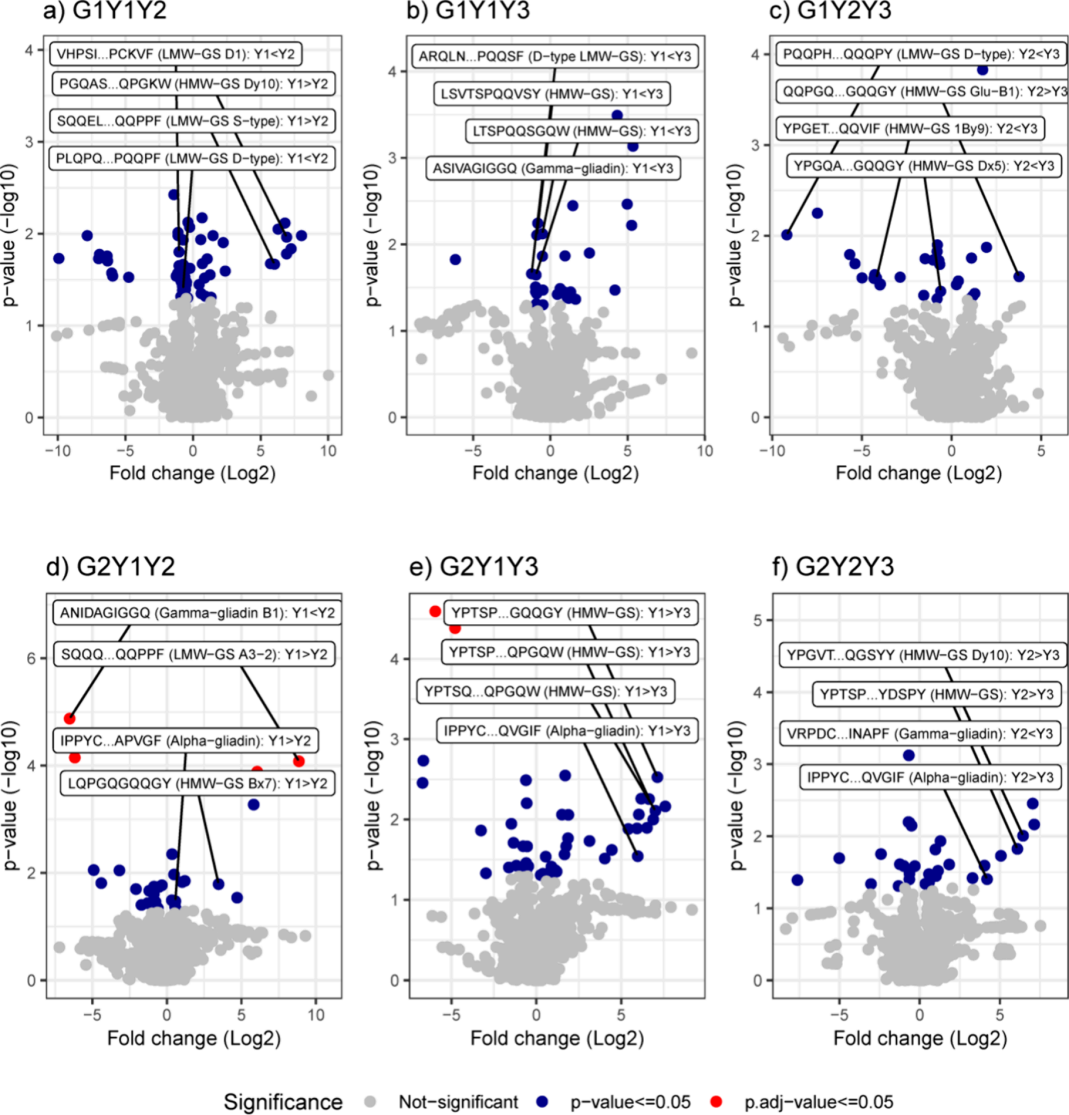
Volcano plots to indicate peptides linked to different proteins from different years and genotypes (Diskett; a–c, and Bumble; d–f). Statistical significance was set at *p* < 0.05 (red dots, *p* adjusted value ≤0.05; Bonferroni correction method; blue dots, *p* value ≤0.05; Student *t* test). G1 and G2 represent genotypes Diskett and Bumble; Y1, Y2, and Y3 represent 2017, 2018, and 2019, R1, R2, and R3 represents replicates 1, 2, and 3, respectively.

Volcano plots to indicate a statistical significance in protein expression versus magnitude of change between the years were compared for peptides originating from HMW-GS, LMW-GS, and gliadins for both genotypes (Figure 5), and *t* test statistical analysis data are shown in Table 1 (Supporting Information, Data File S2). Comparison of protein types and their corresponding peptides, presented mostly by a particular genotype (either G1 or G2) in different growing years, showed a large variation in enrichment levels (Figure 5 and Table 1). For Diskett, comparison between 2017 and 2018 showed that HMW-GS (Dy10) and various types of serpins were greatly present in year 2017, whereas gamma-gliadins and some of LMW-GS were highly present in year 2018 (Table 1 and Figure 5a). For G1, comparing 2017 with 2019, HMW-GS contained higher gamma-gliadins, and LMW-GS (D-type) in year 2019, whereas the amount of serpins was higher in 2017 (Figure 5 and Table 1). In the 2018/2019 comparison, the protein groups HMW-GS (Dx5 and 1By9) and LMW-GS (D-type) were highly enriched in year 2019 (Table 1 and Figure 5c).

For Bumble, comparison between years showed a similar trend as observed in Diskett between 2017 and 2018, where a large impact was observed on LMW-GS (A3-2) and HMW-GS (Bx7) types, as well as on gliadins and serpins, which were more enriched in 2017 (Table 1 and Figure 5d). A comparison between 2017 and 2019 showed that large amounts of enriched groups of HMW-GS, serpins, and alpha-gliadins were present in 2017 compared to 2019, where large amounts of gamma-gliadins were present (Table 1 and Figure 5e). In 2018/2019, HMW-GS (Dy10) and alpha-gliadins were largely present in 2018 and gamma-gliadins were present in 2019, respectively (Table 1 and Figure 5f).

The amount of serpins in the enriched polymeric gluten fraction was relatively high between both genotypes in 2017 compared to years 2018 and 2019, which suggests that colder climate might have contributed to this increase. A general increase in gamma-gliadins was also observed for both genotypes in 2019, which was designated as the high-temperature and precipitation fluctuating year. A comparison of total protein expressed in both genotypes showed that HMW-GS 1Ax1 was present in Diskett in all the years and HMW-GS Dy10 was present in 2017 and 2019, according to LC-MS/MS (Table 1). These particular subunits are associated with gluten strength, protein composition, and dough mixing properties.^52^ A number of peptides found in this study belong to HMW-GS, LMW-GS, serpins, and presence of alpha/beta, gamma, and omega gliadins, which were also confirmed in the proteomics analysis of the polymeric gluten protein fraction in previous studies.^26,53−55^ We can conclude that the main variation between the genotypes studied can be assigned to the polymeric gluten fraction composition and less to the growing environment, as was clearly pointed out by the different results.

In summary, the polymeric gluten fraction is an important quality component, although its polymeric form had to be reduced for LC-MS/MS analysis for comparison of the main proteins and peptides. Hereby, we clearly show that the differentiation is possible by LC-MS/MS on the protein level originating from the genotypic variation and less efficient to evaluate the climate impact and enrichment levels of HMW- and LMW-GS proteins. From polymeric proteins, 7 HMW-GS and 24 LMW-GS were identified in both genotypes, with dominant subunits Dx5 and Dy10, as well as different types of gliadins. In addition, a large number of nongluten-type proteins and enzymes, such as serpins, beta-amylase, and globulins, were also identified, and these might be “trapped” in the polymeric gluten fraction. Diskett showed a more stable and climate-resistant protein pattern in changing climates as compared to Bumble. The use of NEM in this study was applied for differentiation of unbounded free cysteine residues from the incorporated ones in disulfide bridges in HMW-GS and LMW-GS in the digested peptides, which was less successful and should be further explored in a follow-up study. This study concludes that the LC-MS/MS analysis and proteomics approach is suitable for differentiation of wheat genotypes and is less sensitive (only small differences were observed) for evaluation of diverse environmental impacts on the polymeric protein fraction. However, a more thorough combination of several analytical tools may make a compromise and help to develop protein markers for identifying HMW-GS and LMW-GS (and gliadins), which might be a promising tool in future wheat breeding programs.

## Supporting Information Available

Precipitation (mm) and temperature (°C) during the wheat growing period under 2017, 2018, and 2019 (in comparison, and SDS-PAGE of HMW-GS composition of total gluten protein extracted from Diskett and Bumble (PDF)
LFQ intensities of all detected proteins (sheet: proteinGroups) and peptides (sheet: peptides) (XLSX)
*t*-Test results of all the peptides along with their LFQ intensities (XLSX)
