# Supplementary material for: Polymeric Gluten Proteins as Climate-Resilient Markers of Quality: Can LC-MS/MS Provide Valuable Information about Spring Wheat Grown in Diverse Climates?
Source: J Agric Food Chem. 2025 Jan 9;73(3):1844–54. doi: 10.1021/acs.jafc.4c10789 (PMC11760153; doi:10.1021/acs.jafc.4c10789)
Supplement: Supplementary file 1 — jf4c10789_si_001.pdf [file jf4c10789_si_001.pdf]

Polymeric gluten proteins as climate-resilient markers of quality: Can LC-MS/MS provide valuable information on spring wheat grown in diverse climates?

Sbatie Lama,[a,†] Faraz Muneer,[a,†*] Antoine H.P. America[b], Ramune Kuktaite,[a*]

[a]*Department of Plant Breeding, Swedish University of Agricultural Sciences, Box 190, SE-23422 Lomma, Sweden*

[b]*Wageningen Plant Research, Wageningen University and Research, 6708 PB, Wageningen, The Netherlands*

*Email: ramune.kuktaite@slu.se, faraz.muneer@slu.se

† *S:L. and* †*F.M. contributed equally to this work.*

Supporting information

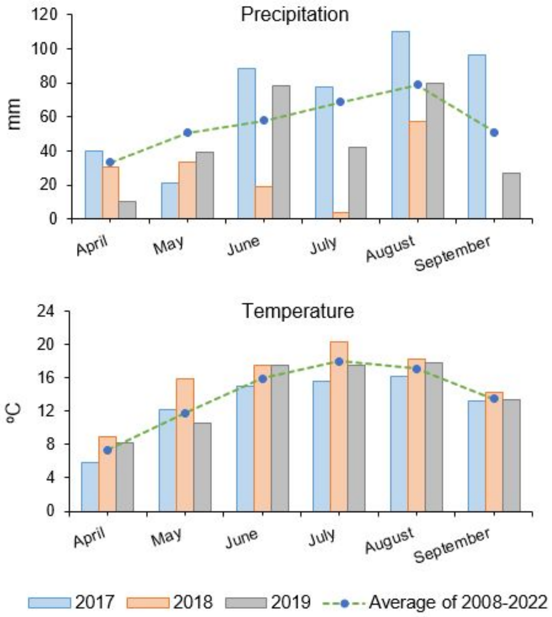

Figure S1. Climatic conditions (precipitation, mm and temperature, °C) during wheat material growing periods under 2017-2019 (staple bars) and the average values of precipitation and temperature (broken line) of 14 years (under the period 2008-2022) in Svalöv, Sweden.

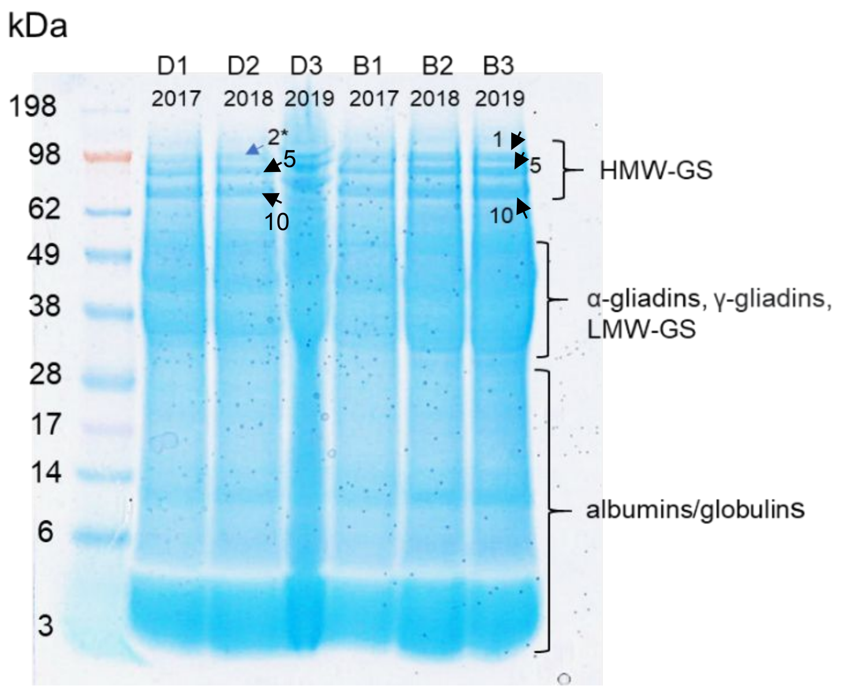

Figure S2. SDS-PAGE gel of total proteins extracted from wheat flour of Diskett (D1, D2 and D3) and Bumble (B1, B2 and B3) (numbers 1-3 showing replicates and 2017, 2018 and 2019 are different years), first to the left shows standard proteins ladder (kDa). Individual HMW-GS 1, 2* and 5+10 are highlighted in both types according to nomenclature of Payne and Lawrence 1983.
